# Molecular functionalization of Ni(OH)_2_ promotes electrosynthesis of adipic acid

**DOI:** 10.1039/d5sc05036g

**Published:** 2025-08-25

**Authors:** Rui Yang, Yuanhao Li, Haonan Xu, Qicheng Zhang, Shufan He, Tao Shen, Xiaobin Fan, Tao Wu, Yifan Sun

**Affiliations:** a Frontiers Science Center for Transformative Molecules, School of Chemistry and Chemical Engineering, State Key Laboratory of Synergistic Chem-Bio Synthesis, Zhangjiang Institute for Advanced Study, Shanghai Jiao Tong University Shanghai 200240 China sunyf@sjtu.edu.cn; b Department of Chemistry, University of Pittsburgh Pittsburgh PA 15213 USA; c School of Chemical Engineering, Dalian University of Technology Dalian Liaoning 116024 China taowu@dlut.edu.cn; d School of Chemical Engineering and Technology, State Key Laboratory of Chemical Engineering, International Joint Laboratory of Low-carbon Chemical Engineering of Ministry of Education, Tianjin University Tianjin 300072 China

## Abstract

Adipic acid is an essential platform molecule for polymer production and is industrially manufactured by thermochemical oxidation of the cyclohexanone/cyclohexanol mixture (KA oil). Alternatively, electrifying provides a green and sustainable route to synthesizing adipic acid, but has been restricted by the low catalytic efficiency. Herein, we report that a nickel hydroxide electrocatalyst functionalized with 4,4′-bipyridine (Bipy-Ni(OH)_2_) delivers a 3-fold greater productivity compared with that of pristine Ni(OH)_2_, achieving an excellent yield (90%) towards efficient adipic acid electrosynthesis. The experimental and molecular dynamics (MD) simulation results show that Bipy serves as a reservoir to accumulate cyclohexanone, which has low solubility in aqueous solutions. Molecular probe analysis coupled with density functional theory (DFT) calculations demonstrates that Bipy functionalization promotes formation of the key intermediate (2-hydroxycyclohexanone) *via* modulating the surface electronic characteristics. A Bipy-Ni(OH)_2_//Ru electro-reforming system in a two-electrode configuration was further constructed to enable concurrent hydrogen and adipate production, revealing its potential for practical applications. Our report demonstrates the efficacy of grafting judicious ligands to electrocatalysts to harness mass transfer and optimize active sites, and the insights can be useful for electrooxidation of a wider scope of organic molecules.

## Abstract 2

Functionalizing Ni(OH)_2_ with 4,4′-bipyridine boosts adipic acid electrosynthesis by threefold, enabled by cyclohexanone enrichment and electronic optimization of the nickel sites.

## Graphical abstract

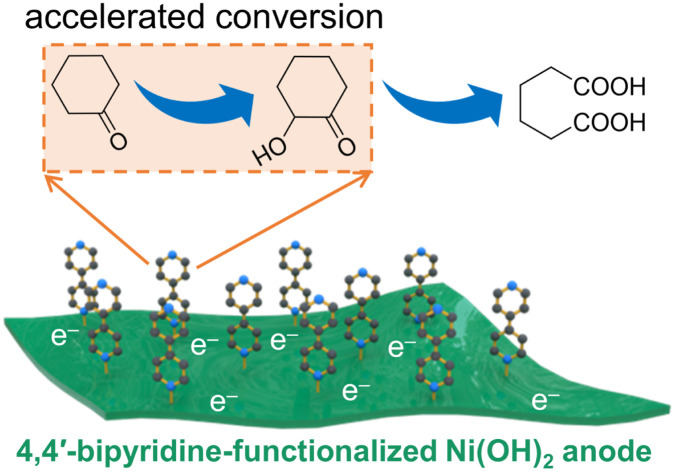

## Introduction

**Scheme 1:**
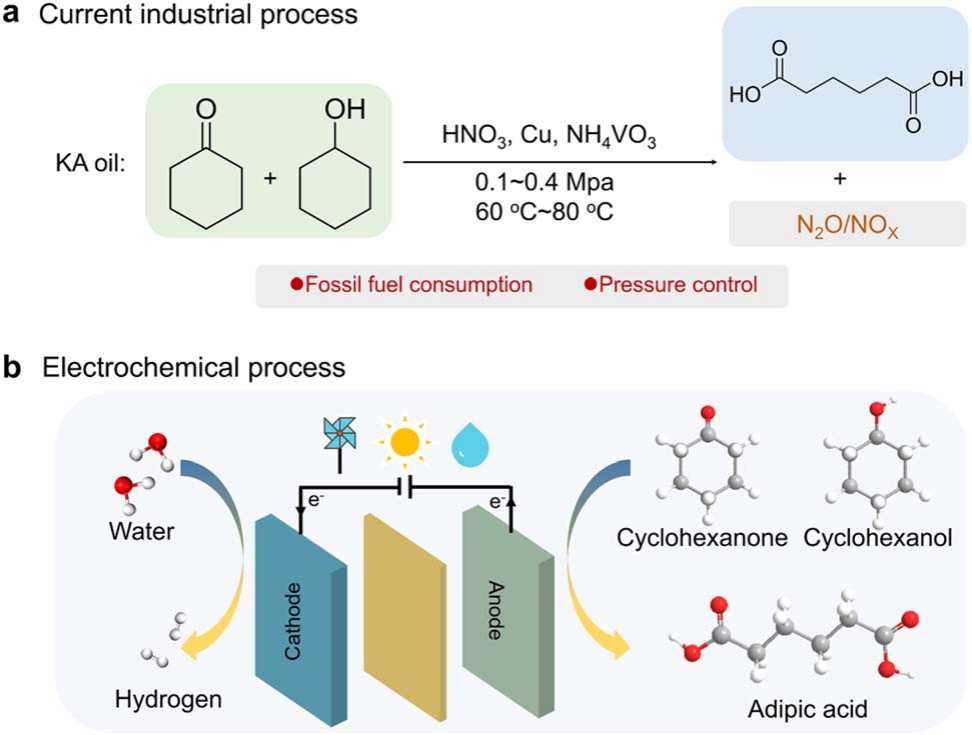
Illustration of the (a) thermochemical and (b) proposed electrochemical routes of KA oil oxidation to produce adipic acid.

Adipic acid, as a crucial dicarboxylic acid, is widely used in the production of polymers, lubricants, pharmaceuticals, food additives and plasticizers, the market size of which is projected to reach over USD 18.2 billion by 2030, particularly as the key building block of nylon-66.^1–3^ Currently, nearly 95% of adipic acid is industrially produced by oxidation of the cyclohexanone/cyclohexanol mixture (KA oil) that involves the use of 50–60% nitric acid as the strong oxidant and a Cu–V compound as the catalyst at elevated pressures and temperatures (Scheme 1a).^4^ This process inevitably generates a large mass of greenhouse gases that calls for elaborate post-treatments. Specifically, for every 10 t of adipic acid manufactured, almost 3 t of noxious nitrous oxide (N_2_O) with atmospheric heat-absorption capacity ∼300 times larger than that of CO_2_ are released.^5^ Therefore, it is critical to develop a green and sustainable alternative for adipic acid production.

Electrocatalytic oxidation provides a competitive route for transforming KA oil into adipic acid under mild conditions using water as the oxygen source and renewable electricity as input energy, meanwhile being coupled with energy-saving H_2_ generation at cathodes (Scheme 1b). The earliest study of electro-reforming adipate can be traced back to the work of Lyalin and Petrosyan in 2004, in which an adipate yield of 52% was obtained over Ni electrodes by cyclohexanone oxidation.^6^ Yi *et al.* later demonstrated that the active sites are NiOOH.^7^ Since then, great endeavors have been made to develop efficient Ni-based electrocatalysts to improve the yield and (or) selectivity of adipic acid. Strategies such as metal doping (Cu, V, Fe, and Mn)^8–11^ and defect regulation^12^ have been reported to improve the yield and/or selectivity of adipate with Ni-based catalysts. For example, O_V_–NiOOH is reported to facilitate the conversion of cyclohexanone (10 mM) to adipic acid benefiting from facile formation of *OOH on O_V_ sites.^12^ In another study, Duan and colleagues intercalated sodium dodecyl sulfonate (SDS) into bulk Ni(OH)_2_ and constructed a hydrophobic microenvironment to accumulate cyclohexanone, obtaining 84% yield of adipic acid with a 20 mM concentration.^13^ Despite these advances, electrosynthesis of adipic acid suffers from the limited cyclohexanone concentration (typically ≤20 mM), which is far from the industrial high-concentration operation requirements and severely compromises the efficiency and cost benefits for large-scale production.^14,15^ Achieving high adipic acid yield with a high cyclohexanone concentration is still challenging due to the inherent low solubility of cyclohexanone in aqueous solutions. This poses obstacles to the associated mass transfer and adsorption of cyclohexanone on the catalyst surface, resulting in degraded catalytic performances. Besides, the intricate reaction mechanisms of cyclohexanone oxidation, which include different intermediates with multiple electron and proton transfer steps, also obstruct optimization of the active sites to accelerate the conversion kinetics. Therefore, it is imperative to identify the critical step for elevating the catalytic activity and design efficient Ni-based electrocatalysts towards electrosynthesis of adipic acid at elevated cyclohexanone concentrations.

Molecular functionalization poses a potentially intriguing approach for accessing efficient electrocatalysts for producing adipic acid. Compared with metal doping and defect regulation mediated by the rigid oxide lattice, a wider scope of molecular ligands with tailorable electronic and geometric traits can be explored.^16,17^ Molecules with heteroatoms like nitrogen, oxygen and sulfur can readily bind with active sites and fine-tune the reaction pathway that leads to higher catalytic activity and selectivity.^18–20^ However, the influence of molecular functionalization on the structural and electronic properties of Ni(OH)_2_ and the reaction kinetics of cyclohexanone oxidation remains elusive.

Herein, we report 4,4′-bipyridine-functionalized Ni(OH)_2_ (Bipy-Ni(OH)_2_) electrocatalysts for efficient conversion of cyclohexanone (100 mM) to adipic acid. Bipy-Ni(OH)_2_ results in an excellent yield (90%) and selectivity (91%) towards adipic acid production, achieving a three-fold increase in productivity relative to that of bare Ni(OH)_2_. The Bipy molecules grafted on Ni(OH)_2_ enable enrichment of cyclohexanone in NaOH aqueous solutions for subsequent conversions. Mechanistic studies reveal that the cyclohexanone conversion follows hydroxylation at the β site and the subsequent selective C_α_–C_β_ cleavage pathway, where Bipy functions as an electronic modifier to promote formation of the key intermediate, 2-hydroxycyclohexanone. In a two-electrode system, the Bipy-Ni(OH)_2_ catalyst delivers 84% yield and 89% selectivity for adipate by KA oil conversion at the anode, along with clean H_2_ production at the cathode, proving its prominent potential for practical applications.

## Results and discussion

**Fig. 1:**
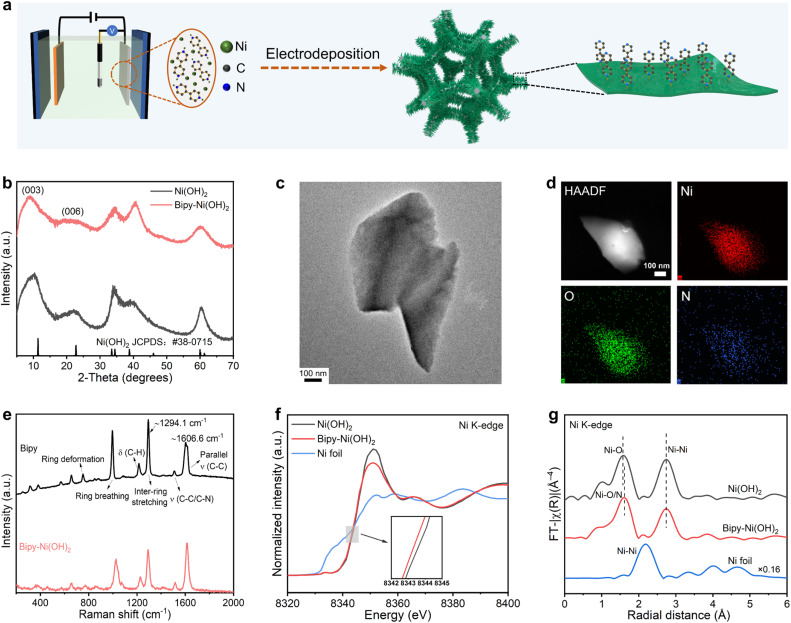
(a) Schematic illustration for the preparation of the Bipy-Ni(OH)_2_ catalyst. (b) XRD patterns of the Bipy-Ni(OH)_2_ and Ni(OH)_2_, and the α-Ni(OH)_2_ (JCPDS: no. 38-0715) reference pattern. (c) TEM image of Bipy-Ni(OH)_2_. (d) HAADF-STEM image and corresponding STEM-EDS element maps of Bipy-Ni(OH)_2_, indicating a uniform distribution of Ni, O and N. (e) Raman spectra of Bipy and Bipy-Ni(OH)_2_. (f) Normalized XANES spectra and (g) *k*^3^-weighted EXAFS profiles of Bipy-Ni(OH)_2_, Ni(OH)_2_ and the Ni foil reference.

N-conjugated ligands were selected because of their coordination capability with the Ni site *via* the nitrogen heteroatom and their rigid conjugated structure that may enhance molecular stability. We synthesized pristine and Bipy-functionalized Ni(OH)_2_ catalysts through a facile electrodeposition method (Fig. 1a). The reduction of nitrate and water at the cathode generates a high hydroxide concentration gradient at the electrode–electrolyte interface and produces Ni(OH)_2_. 4,4′-Bipyridine was added to prepare Bipy-Ni(OH)_2_, which consists of Ni^2+^ species coordinated with both hydroxyl and nitrogen-terminated Bipy molecules. Fig. 1b shows the powder X-ray diffraction (XRD) data for the as-deposited Ni(OH)_2_ and Bipy-Ni(OH)_2_, as well as the α-Ni(OH)_2_ (JCPDS: no 38-0715) reference pattern. Bipy-Ni(OH)_2_ features an almost identical XRD pattern to Ni(OH)_2_, demonstrating the retained hydroxide lattice upon Bipy functionalization. The transmission electron microscope (TEM) image shows that Bipy-Ni(OH)_2_ displays an irregular plate-like morphology (Fig. 1c). As displayed in Fig. 1d, the scanning transmission electron microscopy-energy dispersive spectroscopy (STEM-EDS) element maps for Bipy-Ni(OH)_2_ and uniform distribution of the Ni, O and N signals highlight homogeneity of Bipy functionalization throughout Ni(OH)_2_. Spectroscopic characterization tools were applied to elucidate the coordination environment of Ni(OH)_2_ and Bipy-Ni(OH)_2_. According to the Raman spectra (Fig. 1e), a dramatic change in the intensity ratio of peaks located at ∼1294.1 cm^−1^ and ∼1606.6 cm^−1^ was found with the isolated Bipy molecules and Bipy-Ni(OH)_2_, demonstrating the strong coordination between Bipy and Ni(OH)_2_ (Fig. S1).^21^ To probe the electronic structures of Ni after introduction of Bipy, X-ray absorption spectroscopy (XAS) was carried out. Fig. 1f shows the Ni K-edge X-ray absorption near-edge structure (XANES) spectra of the as-synthesized pristine Ni(OH)_2_, Bipy-Ni(OH)_2_ and Ni foil reference. A slight shift of photon energy to lower energy in Bipy-Ni(OH)_2_ was observed at half height compared with that of pristine Ni(OH)_2_, indicating a lower Ni oxidation state in Bipy-Ni(OH)_2_. The shift position of the adsorption edge for the Ni K-edge manifests that Bipy functions as an electronic modifier for Ni(OH)_2_, which is also supported by the X-ray photoelectron spectroscopy (XPS) results (Fig. S2). The corresponding *k*^3^-weighted Fourier transform (FT) Ni K-edge extended X-ray absorption fine structure (EXAFS) spectra are shown in Fig. 1g. The two major peaks of Ni(OH)_2_ were assigned to the Ni–O and Ni–Ni single-scattering paths, respectively. For Bipy-Ni(OH)_2_, the first peak is ascribed to the mixing of Ni–O/N single scattering owing to the indistinguishability of the neighboring elements. Moreover, the peak marginally shifts towards larger distances relative to the Ni–O contribution of the pristine Ni(OH)_2_ sample, due to the slightly larger radius of nitrogen atoms compared with that of oxygen atoms.^22,23^

**Fig. 2:**
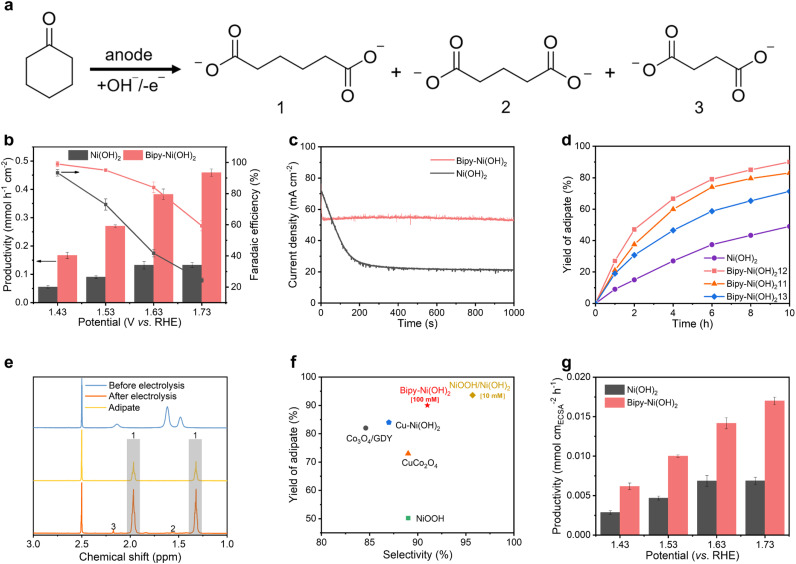
(a) Reaction scheme for the anodic oxidation of cyclohexanone to adipate (1), glutarate (2) and succinate (3). (b) Adipic acid productivity (bar chart, left axis) and FE (line graph, right axis) over Bipy-Ni(OH)_2_ and Ni(OH)_2_ at different potentials in 1.5 M NaOH with 100 mM cyclohexanone. (c) *i*–*t* curves of Bipy-Ni(OH)_2_ and Ni(OH)_2_ at 1.53 V *vs.* RHE in 1.5 M NaOH with 100 mM cyclohexanone. (d) Yield of adipate as a function of reaction time over Ni(OH)_2_ with different Bipy contents at 1.53 V *vs.* RHE in 1.5 M NaOH with 100 mM cyclohexanone. Bipy-Ni(OH)_2_-11, Bipy-Ni(OH)_2_-12, and Bipy-Ni(OH)_2_-13 denote the mole ratio between Bipy and Ni for the electrodeposition as 1 : 1, 1 : 2 and 1 : 3, respectively. (e) ^1^H NMR spectra of the sample before and after electrolysis of Bipy-Ni(OH)_2_ at 1.53 V *vs.* RHE, respectively, and pure adipate for comparison. (f) Comparison of the yield and selectivity of adipate of Bipy-Ni(OH)_2_ with those in previous reports (Table S1). (g) Adipate productivity of Bipy-Ni(OH)_2_ and Ni(OH)_2_ by normalization with ECSA.

Fig. 2 summarizes the catalytic performance of Bipy-Ni(OH)_2_ and Ni(OH)_2_ towards adipate electrosynthesis from cyclohexanone (100 mM). As depicted in Fig. 2b, Bipy-Ni(OH)_2_ exhibits an adipate productivity of 270 μmol cm^−2^ h^−1^ with 95% faradaic efficiency (FE) at 1.53 V *vs.* RHE. By sharp contrast, bare Ni(OH)_2_ delivers one third of adipate productivity (90 μmol cm^−2^ h^−1^) with 73% FE, and no activity was observed for the bare Bipy molecule (Fig. S3), collectively highlighting the promotional effect of Bipy functionalization in adipate electrosynthesis. Besides, Bipy-Ni(OH)_2_ exhibits progressively increased productivities of adipate with increased potentials from 1.43 V to 1.73 V, accompanied by a decrease in FE, which is mainly due to the simultaneous promotion of the competing oxygen evolution reaction (OER). For Ni(OH)_2_, FE decreases when increasing the potential from 1.43 V to 1.73 V, while the adipate productivity increases and reaches a plateau above 1.63 V. We speculate that this difference can be caused by the low capability of pristine Ni(OH)_2_ to accumulate cyclohexanone, resulting in a restrictive mass transfer process. In consequence, the added input electricity only promotes the competing OER process. The chronoamperometry results in Fig. 2c show that the current density of Ni(OH)_2_ experiences a rapid decrease within ∼200 s, which may be due to the fast consumption of cyclohexanone over the Ni(OH)_2_ surface. In comparison, Bipy-Ni(OH)_2_ exhibits a much larger current density, implying its better catalysis performance for cyclohexanone oxidation.

In addition to productivity, the yield of adipate under high conversion of cyclohexanone was also evaluated. As shown in Fig. 2d, the content of Bipy has a notable impact on the yield of adipate. A higher or lower Bipy content results in a lower yield, revealing a trade-off between effective molecular functionalization and excessive functionalization blocking the active sites.^24,25^ The optimized sample exhibits a 90% adipate yield after 10 h electrolysis, highly exceeding that of Ni(OH)_2_ (49%). The minor by-products include succinic acid and glutaric acid, as determined by the nuclear magnetic resonance (NMR) data for Bipy-Ni(OH)_2_ (Fig. 2a and e). The selectivity of adipate over Bipy-Ni(OH)_2_ is calculated to be 91%. Compared with previous reports on adipic acid electrosynthesis, Bipy-Ni(OH)_2_ offers superior catalytic performance regarding yield, selectivity, and the concentration of cyclohexanone (Fig. 2f and Table S1). The electrochemical active surface areas (ECSAs) of Ni(OH)_2_ and Bipy-Ni(OH)_2_ were measured to identify if the enhanced electrocatalytic performance originates from the increase of the surface area due to the ligand-directed nanostructuring or the inherent promotional effect of Bipy functionalization (Fig. S4). Although Bipy-Ni(OH)_2_ features larger ECSA compared with that of Ni(OH)_2_, the ECSA-normalized adipate productivity of Bipy-Ni(OH)_2_ is still higher than that of Ni(OH)_2_ over potentials ranging from 1.43 V to 1.73 V, highlighting enhancement in the intrinsic catalytic performance of Ni(OH)_2_*via* Bipy functionalization (Fig. 2g).^26,27^ In addition to Ni(OH)_2_, Bipy also exhibits evident promotional effects for other Ni-based LDHs regarding adipate yield and FE (Fig. S5).

**Fig. 3:**
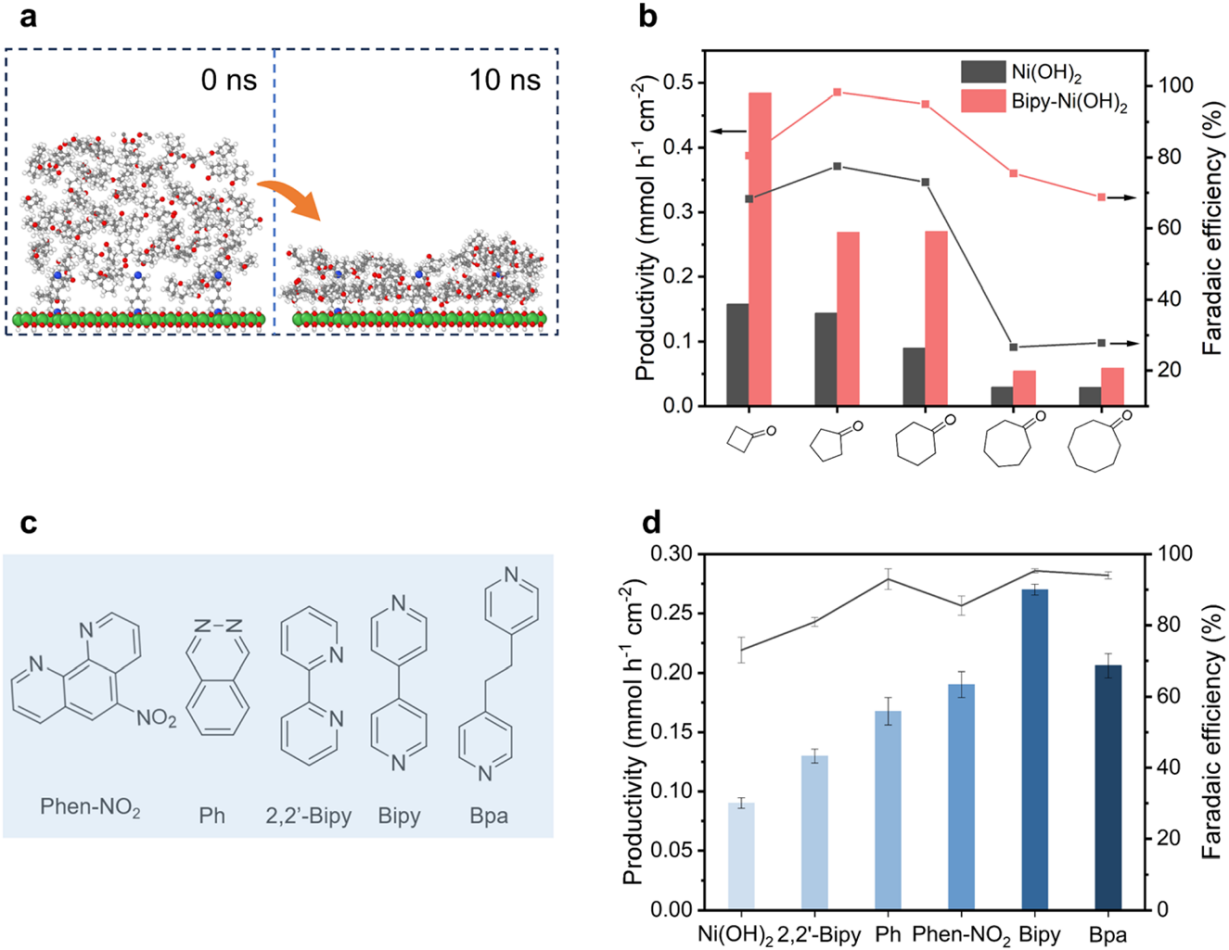
(a) The snapshots from time-dependent MD simulations showing the spatial distribution of cyclohexanone molecules near the Bipy-Ni(OH)_2_ surfaces. (b) The productivity and corresponding FE of products over Bipy-Ni(OH)_2_ and Ni(OH)_2_ with C_4_–C_8_ cyclic ketones with low aqueous solubility at 1.53 V *vs.* RHE in 1.5 M NaOH with 100 mM substrate. (c) Structural illustration of various *N*,*N*-containing molecular ligands. (d) The productivity and corresponding FE of adipic acid electrosynthesis over bare Ni(OH)_2_ and functionalized Ni(OH)_2_ with different N,N-containing ligands at 1.53 V *vs.* RHE in 1.5 M NaOH with 100 mM cyclohexanone.

To elucidate the enrichment behavior of cyclohexanone molecules over different catalyst surfaces, time-dependent molecular dynamics (MD) simulations were conducted.^28^ As shown in Fig. S6, cyclohexanone molecules exhibit low affinity for the Ni(OH)_2_ surface, leading to their dispersion (random or aggregated) within the bulk electrolyte. In contrast, nearly all cyclohexanone molecules move towards and accumulate near the Bipy-Ni(OH)_2_ surface (Fig. 3a). This result indicates that the modified catalyst facilitates the enrichment of cyclohexanone molecules. This enrichment function of Bipy can be further extended to the electrooxidation of a wider array of substrate molecules with limited aqueous solubilities. As displayed in Fig. 3b, for the electrooxidation of C_4_–C_8_ cyclic ketone molecules, Bipy-Ni(OH)_2_ exhibits an observable promotional effect for both productivity and FE of the corresponding products (Table S2). Additional *N*,*N*-containing conjugate ligands including 5-nitro-1,10-phenanthroline (Phen-NO_2_), phthalazine (Ph), 2,2′-bipyridine (2,2′-Bipy) and 1,2-bis(4-pyridyl)ethane (Bpa) were used to functionalize Ni(OH)_2_ (Fig. 3c), denoted as Phen–NO_2_–Ni(OH)_2_, Ph-Ni(OH)_2_, 2,2′-Bipy-Ni(OH)_2_, and Bpa-Ni(OH)_2_, respectively. The successful syntheses of these samples were confirmed by Raman spectroscopy (Fig. S7). All these samples exhibit evident promotional effects both for adipate productivity and FE compared with those of pristine Ni(OH)_2_ (Fig. 3d), demonstrating universality of the molecular functionalization strategy in improving the catalytic performance of Ni(OH)_2_ (Fig. S8). Besides, under high conversion, the ligand-facilitated effect is also retained. As shown in Fig. S9, after 10 h of electrocatalysis, the yield of adipate obtained from these molecule-functionalized Ni(OH)_2_ samples is still higher than that from pristine Ni(OH)_2_.

Now we have demonstrated the reactant accumulation capability of Bipy functionalization and its promotional effect on adipate electrosynthesis. In order to investigate the other roles of Bipy functionalization, the primary task is to pinpoint the reaction path in our system. Industrially, cyclohexanone and cyclohexanol mixtures serve as crude materials to produce adipate. Controlled experiments were performed to understand the difference between cyclohexanone and cyclohexanol upon electrooxidation. The NMR results show that cyclohexanol first undergoes a 2e^−^ oxidation step to generate the cyclohexanone intermediate, which is further oxidized to adipate *via* C–C cleavage. This pathway is the same for both Ni(OH)_2_ and Bipy-Ni(OH)_2_ (Fig. S10a). After 14 h of electrolysis, 88.6% and 46.1% yields of adipate can be obtained over the Bipy-Ni(OH)_2_ and Ni(OH)_2_ catalysts (Fig. S10b). To evidence the transformation process of cyclohexanol to cyclohexanone before obtaining adipate, 1-methylcyclohexanol was chosen as the substrate molecule given its incapability of being oxidized to ketone. As a result, there is no obvious change in 1-methylcyclohexanol before and after electrolysis (Fig. S11). This directs us to focus on the conversion process from cyclohexanone to adipate.

**Fig. 4:**
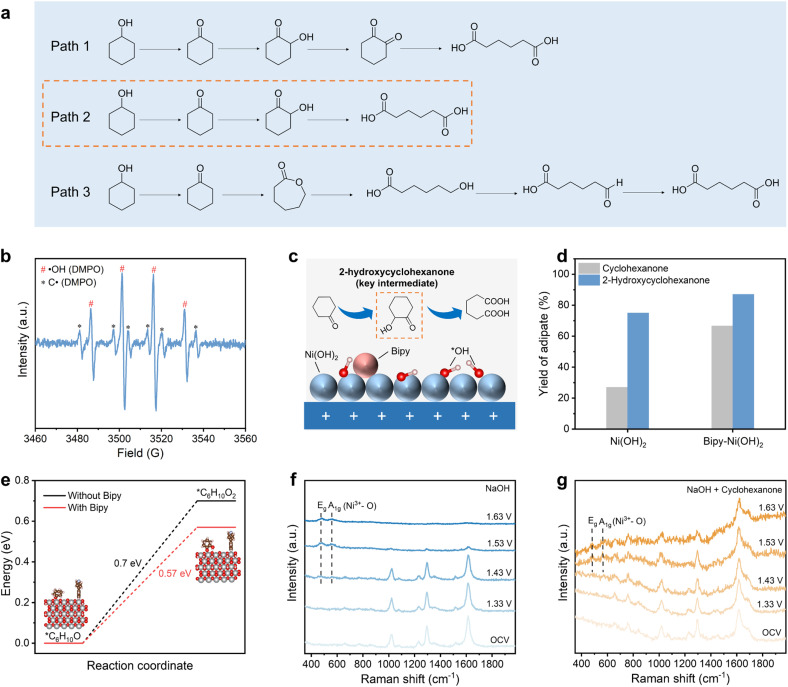
(a) Possible reaction pathways of cyclohexanone oxidation to adipic acid in the literature. (b) EPR signal over Bipy-Ni(OH)_2_ captured in 1.5 M NaOH with 0.1 M cyclohexanone. (c) Schematic of Path 2 with 2-hydroxycyclohexanone as the key intermediate. (d) Yield of adipate electrosynthesis using cyclohexanone and 2-hydroxycyclohexanone as the substrate molecule after 4 h of electrolysis (electrolyte composition: 100 mM substrate molecule + 1.5 M NaOH). (e) DFT-calculated energy diagram of cyclohexanone to 2-hydroxycyclohexanone over the pristine and Bipy-functionalized Ni(OH)_2_ surface. The nickel, oxygen, carbon, hydrogen and nitrogen atoms are in grey, red, brown, pink and blue, respectively. (f) *In situ* Raman spectra of Bipy-Ni(OH)_2_ in the 1.5 M NaOH aqueous solution without and (g) with cyclohexanone.

The above results together with previous reports point out three possible reaction pathways for cyclohexanol oxidation (Fig. 4a). The first path involves 1,2-cyclohexanedione as a crucial intermediate for the following oxidative C–C cleavage between the two neighboring carbonyl groups to produce adipate (Path 1).^29^ The second path indicates that cyclohexanone undergoes hydroxylation at the β site and C–C cleavage before dione formation^8,9^ (Path 2). In the third one, cyclohexanone is oxidized to form the ε-caprolactone intermediate by the Baeyer–Villiger oxidation and ε-caprolactone undergoes subsequent hydrolysis and oxidation to produce adipate (Path 3).^12^ We first applied 1,2-cyclohexanedione as the substrate molecule which mainly results in glutarate, thus ruling out Path 1. Furthermore, electrolysis of 1,3-cyclohexanedione and 1,4-cyclohexanedione leads to formation of a mixture of glutarate and succinate, and succinate as the major product (Fig. S12 and S13), respectively, thus excluding dione being the key intermediate. To distinguish between Path 2 and Path 3, we conducted electron paramagnetic resonance (EPR) spectroscopy using 5,5-dimethyl-1-pyrroline N-oxide (DMPO) as the capturer to identify the as-formed radical species during the electro-oxidation process.^30^ As shown in Fig. 4b, hydroxyl (⋅OH) and carbon-centered radicals (C⋅) were observed in our case. Only ⋅OH was detected in the absence of cyclohexanone (Fig. S14), suggesting that C⋅ originates from cyclohexanone. This implies that cyclohexanone first undergoes β-H elimination to form C⋅ and subsequently yields 2-hydroxycyclohexanone through oxygenation by ⋅OH. Hydroperoxyl radicals (⋅OOH), which are absent in our case, would be expected following Path 3. Therefore, Path 2 involving 2-hydroxycyclohexanone formation is identified as the major pathway in our case (Fig. 4c). To verify whether Bipy functionalization also influences the following oxidation process, we applied 2-hydroxycyclohexanone as the starting electrocatalysis material. For the 4 h electrolysis, the adipate yield for the electrooxidation of 2-hydroxycyclohexanone using Ni(OH)_2_ and Bipy-Ni(OH)_2_ is 75% and 87%, respectively, both higher than that using cyclohexanone as the substrate (27% for Ni(OH)_2_ and 66.6% for Bipy-Ni(OH)_2_ (Fig. 4d). The notably faster transformation speed indicates that the breaking of C_α_–C_β_ becomes feasible once C_α_ and C_β_ reach a high oxidation state.^8^ The promotional effect of Bipy-Ni(OH)_2_ on the oxidation of 2-hydroxycyclohexanone may be due to the enhanced oxidizing power resulting from electronic modification induced by Bipy functionalization. This is further supported by the oxidation result of 1,6-hexanediol (Fig. S15).

The above results suggest that the conversion from 2-hydroxycyclohexanone to adipate in Path 2 is favorable. We thereby reason that the way towards the 2-hydroxycyclohexanone formation is the key step that leads to distinct catalytic performances of Ni(OH)_2_ and Bipy-Ni(OH)_2_. Density functional theory (DFT) calculations were adopted to compare the energetic barrier from cyclohexanone to 2-hydroxycyclohexanone over the pristine and functionalized Ni(OH)_2_ catalyst. As displayed in Fig. 4e and S16, Ni(OH)_2_ exhibits a 0.7 eV barrier for pristine Ni(OH)_2_, while the value decreases to 0.57 eV for Bipy-Ni(OH)_2_ (barrier refers to the minima of the different products coordinated to the different Ni sites). This implies that the electronic modulation of Bipy on Ni(OH)_2_ facilitates the formation of 2-hydroxycyclohexanone.^31^ Based on this experimental and computational evidence, we attribute the crucial role of Bipy functionalization to facilitating production of the key intermediate (2-hydroxycyclohexanone) *via* electronic regulations.

Besides, to gain insights into the structure transformation and clarify the actual active sites, we employed *in situ* Raman spectroscopy (Fig. S17). As shown in Fig. 4f, upon applying anodic potentials in 1.5 M NaOH, evolution of the E_g_ bending and A_1g_ stretching mode of Ni^3+^–O was observed, along with leaching of the Bipy molecule, evidencing a total restructuring from Ni(OH)_2_ to NiOOH under OER conditions,^32,33^ while in 0.1 M cyclohexanone, with the increase of potentials, no obvious Bipy dissolution is observed (Fig. 4g). The difference between the Raman spectra at the open-circuit voltage (OCV) in NaOH (1.5 M) and that in NaOH (1.5 M) + cyclohexanone (100 mM) is due to the overlap of the peak features of cyclohexanone and Bipy.^34^ Besides, the Ni^3+^–O signals under cyclohexanone oxidation conditions emerged at higher voltage compared with that of the OER conditions. This implies that the Ni^3+^–O species serve as redox mediators during the cyclohexanone oxidation process, causing partial consumption of Ni^3+^–O species.^9,35–37^

**Fig. 5:**
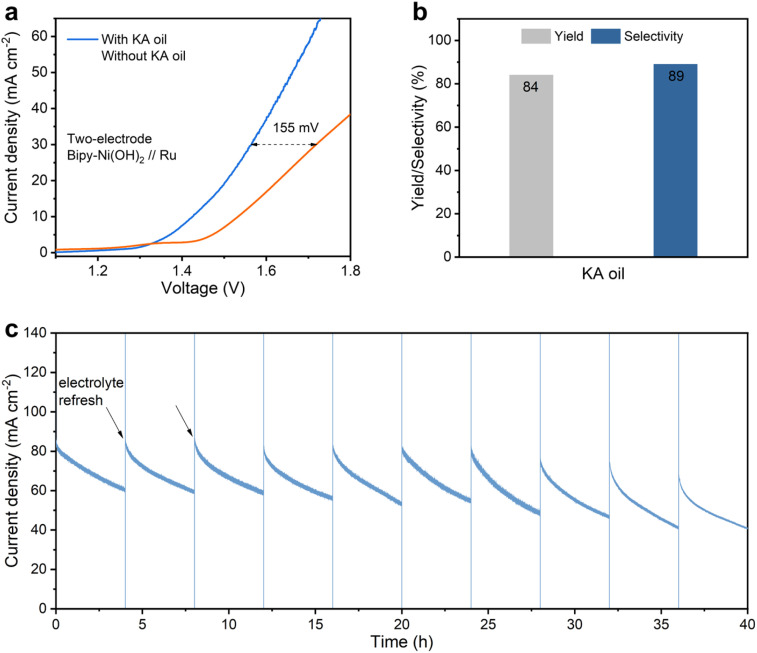
(a) Linear sweep voltammetry (LSV) curves of the Bipy-Ni(OH)_2_//Ru couple in 1.5 M NaOH with and without KA oil (3 : 2 of cyclohexanol and cyclohexanone) at a scan rate of 5 mV s^−1^. (b) Yield and selectivity of adipate using the Bipy-Ni(OH)_2_//Ru couple with a two-electrode configuration at 1.8 V. (c) The long-term *i*–*t* curve of KA oil oxidation over Bipy-Ni(OH)_2_//Ru at 1.80 V, with the electrolyte (1.5 M NaOH with KA oil) refreshed every 4 h.

Towards practical applications, we coupled the Bipy-Ni(OH)_2_ catalyst with a Ru hydrogen evolution catalyst to construct a two-electrode electro-reforming system for concurrent hydrogen and adipate production at the cathode and anode, respectively. In order to simulate industrial scenarios, KA oil composed of cyclohexanol and cyclohexanone (3 : 2) was chosen as the starting material. As shown in Fig. 5a, the system with KA oil delivers a more energy-saving process compared with that of the water splitting one with a nearly 155 mV potential decrease at 30 mA cm^−2^, corresponding to ∼9% energy saving. Favorable kinetics with a smaller Tafel slope (166.1 mV dec^−1^) was also achieved with the KA oil system relative to that of the water splitting process (199.4 mV dec^−1^, Fig. S18). For the electrolysis of KA oil at 1.8 V, an 84% yield and 89% selectivity of adipate acid at the anode and pure H_2_ generated at the cathode can be achieved (Fig. 5b and S19a), demonstrating the practical potential of Bipy-Ni(OH)_2_. The stability of the Bipy-Ni(OH)_2_//Ru catalyst was further evaluated with the long-term (40 h) electrolysis measurement (Fig. 5c). For each 4 h cycle, the current density gradually decreases due to consumption of the reactant (KA oil) and instantly recovers upon the electrolyte refresh. The TON was estimated to be 535 per Ni-site (Fig. S19b). A slight decrease in current density over the long term indicated reasonable stability of the electrode material.

## Conclusions

In summary, we achieved efficient electrooxidation of KA oil to adipate by using Bipy to functionalize the Ni(OH)_2_ catalyst. The tethered Bipy molecules facilitate accumulation of cyclohexanone in the vicinity of the catalysts in aqueous solutions, enabling sufficient supply of reactant molecules transported from the bulk electrolyte. Through electronic interactions, Bipy functionalization also promotes formation of 2-hydroxycyclohexanone, which is experimentally identified as the key intermediate towards efficient adipate production. Collectively, Bipy-Ni(OH)_2_ exhibits 90% yield and 91% selectivity and excellent stability and recyclability towards the electrosynthesis of adipic acid. The practicality of the Bipy-Ni(OH)_2_ catalyst was also demonstrated using a two-electrode electrolysis system, where KA oil was efficiently transformed to produce adipic acid and pure H_2_ at the anode and cathode, respectively.

Our work emphasizes the significance of molecular functionalization of solid electrocatalysts for enhanced performance. The organic–inorganic interface affords a diverse platform for harnessing the mass transfer behavior of reactants and optimizing the metal active sites. It is anticipated that more molecular ligands can be explored as both catalytically tunable modifiers and effective hydrophobic agents for enhanced catalysis. The molecular functionalization strategy can be further extended to supported heterogeneous catalysts, for which the metal–support interactions may be tailored with molecular precision.

## Author contributions

R. Y. carried out the synthesis, characterization and electrocatalytic experiments. Y. L. performed the MD simulations. H. X. and T. W. performed the DFT calculations. Q. Z. and X. F. performed the XAS measurements. S. H. and T. S. synthesized 2-hydroxycyclohexanone for mechanistic studies. Y. S. conceived and directed the project. R. Y., Y. L., T. W. and Y. S. prepared the manuscript.

## Conflicts of interest

There are no conflicts to declare.

## Supplementary Material

SC-OLF-D5SC05036G-s001

## Data Availability

The authors declare that the data supporting the findings of this study, including the characterization result, electrochemical performance and MD and DFT simulation details, are available from the corresponding author, Y. S., upon reasonable request. Supplementary information: experimental and calculation details, additional material characterization and electrosynthesis results, and performance comparison. See DOI: https://doi.org/10.1039/d5sc05036g.
