# Supplementary material for: Molecular functionalization of Ni(OH)2 promotes electrosynthesis of adipic acid
Source: Chem Sci. 2025 Aug 25;16(38):17803–11. doi: 10.1039/d5sc05036g (PMC12409666; doi:10.1039/d5sc05036g)
Supplement: SC-016-D5SC05036G-s001 [file SC-016-D5SC05036G-s001.pdf]

Supplementary Information (SI) for Chemical Science.
This journal is © The Royal Society of Chemistry 2025

Supporting Information

Molecular Functionalization of $Ni(OH)_2$ Promotes Electrosynthesis of Adipic Acid

Rui Yang[a], Yuanhao Li[b], Haonan Xu[c], Qicheng Zhang[d], Shufan He[a], Tao Shen[a], Xiaobin Fan[d], Tao Wu[c]*, Yifan Sun[a]*

[a]Frontiers Science Center for Transformative Molecules, School of Chemistry and Chemical Engineering, Zhangjiang Institute for Advanced Study, Shanghai Jiao Tong University, Shanghai 200240, China.

[b]Department of Chemistry, University of Pittsburgh, Pittsburgh, PA, 15213, USA.

[c]School of Chemical Engineering, Dalian University of Technology, Dalian, Liaoning 116024, China.

[d]School of Chemical Engineering and Technology, State Key Laboratory of Chemical Engineering, International Joint Laboratory of Low-carbon Chemical Engineering of Ministry of Education, Tianjin University, Tianjin, 300072, China.

Chemicals and materials.

All reagents were used as received without further purification. Nickel nitrate hexahydrate ($Ni(NO_3)_2·6H_2O$, 98%) was purchased from Sinopharm Chemical Reagent Co., Ltd. (China). cyclohexanol (> 99.0%), cyclohexanone (99.5%), cyclobutanone (99%), cyclopentanone (99.5%), cycloheptanone (99%), cyclooctenone (97%) sodium hydroxide (NaOH, 98%), 1,2-cyclohexanedione (98%), 1,3-cyclohexanedione (97%), 1,4-cyclohexanedione (98%), 1-methylcyclohexanol (99%), 1,6-hexanediol (99.5%), adipic acid (99.5%), 5-nitro-1,10-phenanthroline (Phen-NO_2, 98%), phthalazine (Ph, 98%), and caprolactone (99%) were purchased from Aladdin. 4,4'-bipyridine (Bipy, 98%), 2,2'-bipyridine (2,2'-Bipy, 99%), and 1,2-bis(4-pyridyl)ethane (Bpa, 97%) were purchased from Shanghai Macklin Biochemical Co., Ltd. Deuterium oxide (D_2O, 99.9 atom%D) was purchased from Beijing InnoChem Science & Technology Co., Ltd. Nitrosobenzene (PhNO, 97%), dimethyl sulfoxide (DMSO, 99.7%, Extra Dry, with molecular sieves) and methanol (MeOH, 99.8%, Extra Dry, with molecular sieves) were purchased from Energy Chemical. Copper(II) sulfate ($CuSO_4$, 98%) was purchased from Bidepharm. L-Proline (98%) was purchased from Leyan. Nickel foam (NF) was purchased from GaossUnion. Ultrapure deionized water (18.2 MΩ/cm, 25 °C) was obtained from the ELGA purification system (China).

As illustrated in Scheme S1, 2-hydroxycyclohexanone was prepared using the following protocol. According to the method reported in the literature,[1] a round-bottomed flask equipped with a stirring bar was charged with nitrosobenzene (1.0 mmol, 1.0 equiv.), L-proline (0.2 mmol, 20 mol%) and DMSO (4 mL). After being stirred for 5 min, cyclohexanone (10 mmol, 10 equiv.) was added to the solution. After 6 h of vigorous stirring, the reaction was quenched by the addition of aqueous NH_4Cl, and the aqueous phase was extracted with EtOAc (3×10 mL). The combined organic layers were washed with brine (30 mL), dried over using anhydrous Na_2SO_4, and then concentrated under reduced pressure. The crude product was purified by flash column chromatography (PE/EtOAc = 8:1) on silica gel to afford the 2-((phenylamino)oxy)cyclohexan-1-one (113 mg, 55%) as a yellow solid.

According to the method reported in the literature,[2] a round-bottomed flask equipped with a stirring bar was charged with 2-((phenylamino)oxy)cyclohexan-1-one (1.0 mmol, 1 equiv.) and dissolved in MeOH (5 mL). After being cooled to 0 °C, $CuSO_4$ (0.3 mmol, 30 mol%) was added to the solution. After stirring for 12 h at 0 °C, the reaction was quenched by the addition of aqueous NH_4Cl, and the aqueous phase was extracted with EtOAc (3×10 mL). The combined organic layers were washed with brine (30 mL) and dried over anhydrous Na_2SO_4, and then concentrated under reduced pressure. The crude product was purified by flash column chromatography (PE/EtOAc = 4:1) on silica gel to afford the 2-hydroxycyclohexanone (68 mg, 60%) as a pale yellow oil.

Materials characterization.

Powder XRD patterns were collected using a ThermoFisher Scientific Equinox 3500 X-ray diffractometer equipped with a Cu Kα X-ray source (λ = 1.5418 Å). TEM, HAADF-STEM images and STEM-EDS data with element maps were acquired using a Tecnai G2 F30 filed emission transmission electron microscopy operated at 300 kV. The Raman

spectroscopy was carried out on Horiba LabRAM Solei with the excitation wavelength of 532 nm. The absorption spectra of Ni K-edge were collected in transmission mode at the BL14W1 station of the Shanghai Synchrotron Radiation Facility. The X-ray absorption data at the Ni K-edge of the samples were recorded at room temperature in the transmission mode using ion chambers at beam line BL14W1 of the Shanghai Synchrotron Radiation Facility (SSRF), China.[3] The station was operated with a Si (111) double crystal monochromator. During the measurement, the synchrotron X-ray was operated at the energy of 3.5 GeV and a current between 150-210 mA. The photon energy was calibrated with the first inflection point of Ni K-edge in the Ni metal foil. The XAS spectra were analyzed using the Athena and Artemis software,[4] and normalized with linear and cubic fits of the pre-edge and post-edge regions, respectively. Electron spin resonance (EPR) was conducted on Bruker A300. X-ray photoelectron spectroscopy (XPS) analysis was made with a Thermo Scientific K-Alpha device. All XPS spectra were corrected using C1s line.

Catalyst electrode preparation.

$Ni(OH)_2$, Bipy-$Ni(OH)_2$, 2,2'-Bipy-$Ni(OH)_2$, Ph-$Ni(OH)_2$, Phen-NO_2-$Ni(OH)_2$ and Bpa-$Ni(OH)_2$ were prepared by electrodeposition with a three-electrode configuration. Typically, 25 mL of 0.2 M $Ni(NO_3)_2$ and 0.1 M ligand molecules with H_2O and N,N-dimethylformamide mixed solution were used as the electrolyte, and Ni foam was used as the working electrode, which was first treated in 3 M HCl and deionized water to remove the oxidative layers. Ag/AgCl electrode and Pt was used as the reference and counter electrode, respectively. The electrodeposition was carried out under a constant cathodic potential of –2 V (vs. Ag/AgCl) for 200 s. The obtained electrode was thoroughly washed with ultra-pure deionized water and dried at 60 °C for subsequent electrochemical tests. Bipy-$Ni(OH)_2$-11, Bipy-$Ni(OH)_2$-12 and Bipy-$Ni(OH)_2$-13 denotes the mole ratio between Bipy and Ni for the electrodeposition as 1:1, 1:2 and 1:3, respectively.

Electrochemical measurement.

The electrochemical studies were performed on a CHI 760E workstation using a standard H-type electrochemical cell with a three-electrode configuration. An Hg/HgO electrode and a Pt sheet was used as the reference electrode and counter electrode, respectively. The as-prepared 1 cm^2 catalysts on the NF directly served as the working electrode. All the measured potentials were converted to the reversible hydrogen electrode (RHE) according to the equation below:

$E_{(RHE)} = E_{(Hg/HgO)} + 0.098 + 0.059 \times pH$

The electrochemical oxidation reaction was conducted in the aqueous solution containing 1.5 M NaOH and 0.1 M cyclohexanone/cyclohexanol. Linear sweep voltammetry (LSV) curves were used for the kinetics study under the scan rates of 5 mV/s. For the electrocatalytic oxidation of cyclohexanone, we applied a constant potential of 1.43/1.53/1.63/1.73 V vs. RHE for a specific reaction time (0-10 h). Electrochemical oxidation of the related substrate molecules serving as molecular probes (cyclohexanone, cyclohexanol, 1,2-cyclohexanedione, 1-methylcyclohexanol, 1,6-hexanediol, 2-hydroxycyclohexanone, and caprolactone) was also carried out in the aqueous solution

with 1.5 M NaOH and 0.1 M of the substrate molecule. All experiments were conducted at room temperature (~25 °C).

C_{dl} was estimated by plotting the $\Delta j = (j_a - j_c)$, where ja and jc anode and cathode current density to scan rate, where the slope is twice that of C_{dl}. The electrochemical active surface area (ECSA) of catalyst was calculated by the following equation:

ECSA = C_{dl}/C_s

Pulsed CA measurements were performed to estimate the numbers of active Ni sites, and the results are shown in Figure S19. The TON value was estimated as the moles of product divided by the moles of Ni sites, N_{Ni} = Q/F (N is the moles of Ni site, Q is integral area, F is Faraday constant, 96485 C mol^{-1}):

$$TON = \frac{0.00838\, mol\, x\, 96485\, C/mol}{1.511\, C} = 535$$

Product analysis.

The obtained products were analyzed by NMR spectroscopy on a Bruker AVANCE NEO (500 MHz) spectrometer. Specifically, 100 μL electrolyte was added to 400 μL D_2O with DMSO as the internal standard. The measured product exists in the form of adipate rather than adipic acid since the product is not acidified.

The mole numbers of the product were calculated by the following equation:

$$n_{\text{adipate}} = \frac{A_{\text{adipate}}\, N_{\text{DMSO}}}{A_{\text{DMSO}}\, N_{\text{adipate}}} \times n_{\text{DMSO}}$$

where $A_{adipate}$ and A_{DMSO} is the integral area of the adipate peak and internal standard DMSO peak, respectively. $N_{adipate}$ and N_{DMSO} denotes the H number of the adipate peak and the DMSO peak, respectively, and n_{DMSO} is the mole number of the DMSO internal standard.

The yield of the adipate was calculated by the following equation:

$$\text{Yield of adipate } (\%) = n_{\text{adipate}} / n_{\text{substrate}} \times 100\%$$

where $n_{adipate}$ is the mole number of adipate, and $n_{substrate}$ is the mole number of the substrate molecule.

The Faraday efficiency (FE) was calculated by the following equation:

$$FE_{\text{adipate}}\, (\%) = \frac{n_{\text{adipate}}\, z_{\text{adipate}}\, F}{Q_{\text{total}}} \times 100\ \%$$

where $z_{adipate}$ = 6 is the number of electrons required to produce an adipate, F is the Faraday constant (96485 C/mol), and Q_{total} is the total charge input.

EPR measurement.

100 μL of electrolyte (1.5 M NaOH with or without 100 mM cyclohexanone), electrolyzed at a constant potential of 1.53 V vs. RHE for 8 min, was taken out and immediately combined with the DMPO spin trap. The mixture was absorbed into capillary tubes and analyzed by EPR spectroscopy (Bruker A300).

In situ Raman measurement.

The in-situ Raman spectra were recorded by Raman spectrometer (Horiba LabRAM Solei) equipped with a 532 nm laser and 50X objective lens equipped with Electrochemical Workstation (Auto lab). The measurement was conducted on an in situ Raman cell (C031-2, Gaoss Union Tech. Co., Ltd.), with the carbon rod as the counter electrode, the Ag/AgCl electrode as the reference electrode and the electrodeposited sample Bipy-$Ni(OH)_2$ as working electrode (electrolyte: 1.5 M NaOH with and without 100 mM cyclohexanone).

Density functional theory calculations.

Spin-polarized density functional theory + U (DFT + U) calculations were performed using the Vienna ab initio simulation package (VASP)[5-7] with the generalized gradient approximation (GGA) and Perdew-Burke-Ernzerhof (PBE) functional[8]. The interaction between core and valence electrons was described using the projector augmented wave (PAW) method[9,10], with a plane wave energy cutoff of 400 eV. Brillouin zone integration was conducted using a k-point grid containing only the gamma point. The convergence criteria for total energy and force were set to 1×10^{-4} eV and 0.05 eV/Å, respectively. To accurately account for the strong localized Coulomb interactions arising from the localization of Ni 3d orbitals, a Hubbard U parameter of 5.0 eV was applied to the Ni 3d states.[11] The topmost atomic layer was fully relaxed, while the remaining layers were fixed. A 15 Å vacuum layer was added along the Z direction to prevent interactions between the periodic images.

The Gibbs free energy change (ΔG) for elementary step was calculated using the computational hydrogen electrode (CHE) model,[12] as detailed below:

$$\Delta G = \Delta E + \Delta E_{ZPE} - T\Delta S$$

where ΔE is the electronic energy contribution directly obtained from density functional theory (DFT) calculations. ΔE_{ZPE} and TΔS represents the contribution of zero-point energy and entropy (at 298.15 K), respectively. For free molecules, these values can be acquired from the NIST Chemistry Webbook,[13] and the values for the intermediates were determined by calculating their vibrational frequencies.

Molecular dynamics calculations.

All molecular dynamics (MD) simulations were performed using the open-source software package LAMMPS[14] and atomic structures were visualized using OVITO.[15] The AMBER GAFF2 force field[16] was used to model interactions among the cyclohexanone molecules, and the coupled interaction strengths at the solid-fluid interface are predicted using the Lorentz-Berthelot mixing rule:[17]

$$U(r_{ij}) = 4\varepsilon\left[\left(\frac{\sigma}{r_{ij}}\right)^{12} - \left(\frac{\sigma}{r_{ij}}\right)^{6}\right]\#$$

where r_{ij} is the interparticle separation between particles *i* and *j*, and σ and *ε* are characteristic length and energy scales of the Lennard-Jones interaction. The coupled interaction strengths at the solid-fluid interface are predicted using the Lorentz-Berthelot mixing rule:[18]

$$\sigma_{S-F} = \frac{\sigma_{S-S} + \sigma_{F-F}}{2}\#$$

$$\varepsilon_{S-F} = \sqrt{\varepsilon_{S-S} * \varepsilon_{F-F}} \#$$

where the subscripts S–F, S–S, and F–F refer to the interaction energy scales at the solid-fluid interface, within the solid ($Ni(OH)_2$), and within the fluid (cyclohexanone molecules), respectively.

The system was first energy-minimized via the gradient descent approach. The Nose-Hoover thermostat[19] was used to maintain the equilibrium temperature at 300 K and periodic boundary conditions were imposed on all three dimensions. The Particle Mesh-Ewald method[20] was used to compute long-range electrostatics within a relative tolerance of $1x10^{-8}$. A cut-off distance of 1 nm was applied to real-space Ewald interactions.

SUPPLEMENTARY FIGURES

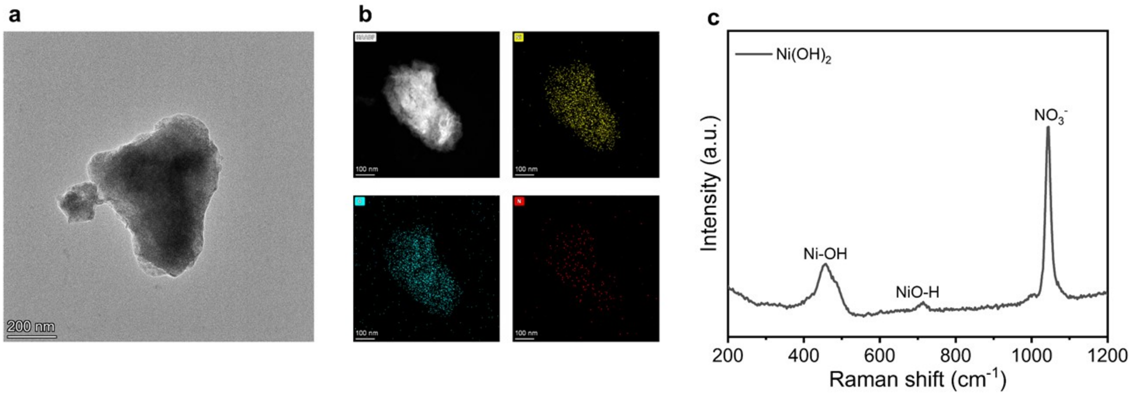

Figure S1. (a) TEM image, (b) HAADF-STEM image and corresponding STEM-EDS element maps, and (c) Raman spectrum of bare $Ni(OH)_2$. The NO_3^- signal in the Raman spectrum is originated from the electrodeposition solution.[21,22]

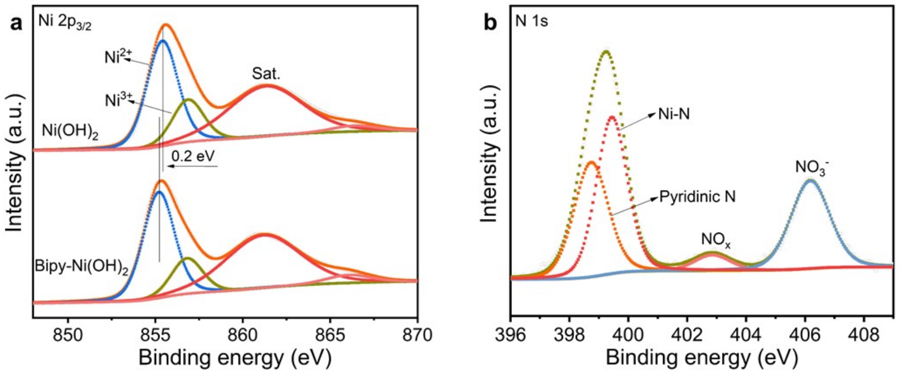

Figure S2. (a) High-resolution XPS spectra showing the Ni $2p_{3/2}$ region for the pristine $Ni(OH)_2$ and Bipy-$Ni(OH)_2$ catalysts. (b) High-resolution XPS spectra showing the N 1s region for Bipy-$Ni(OH)_2$. The binding energy of Ni^{2+} shifts from 855.4 eV for the bare $Ni(OH)_2$ to 855.2 eV for Bipy-$Ni(OH)_2$. In the N 1s XPS spectra of Bipy-Ni(OH), the peaks at 399.4 and 398.7 eV were attributed to the Ni-N bond and pyridine N, respectively, confirming the coordination of BiPy with Ni.[23,24] The additional peaks were attributed to N-O species,[25] which were likely originated from the electrodeposition solution.

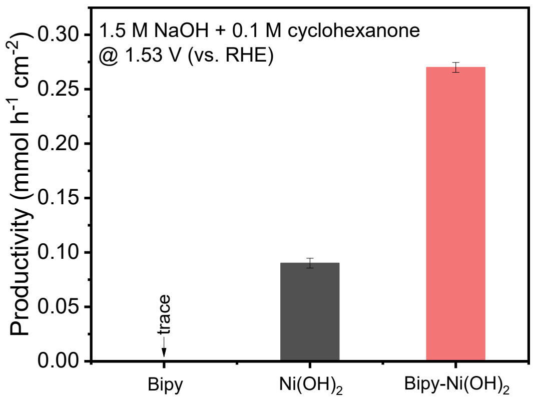

Figure S3. Adipic acid productivity using different catalysts.

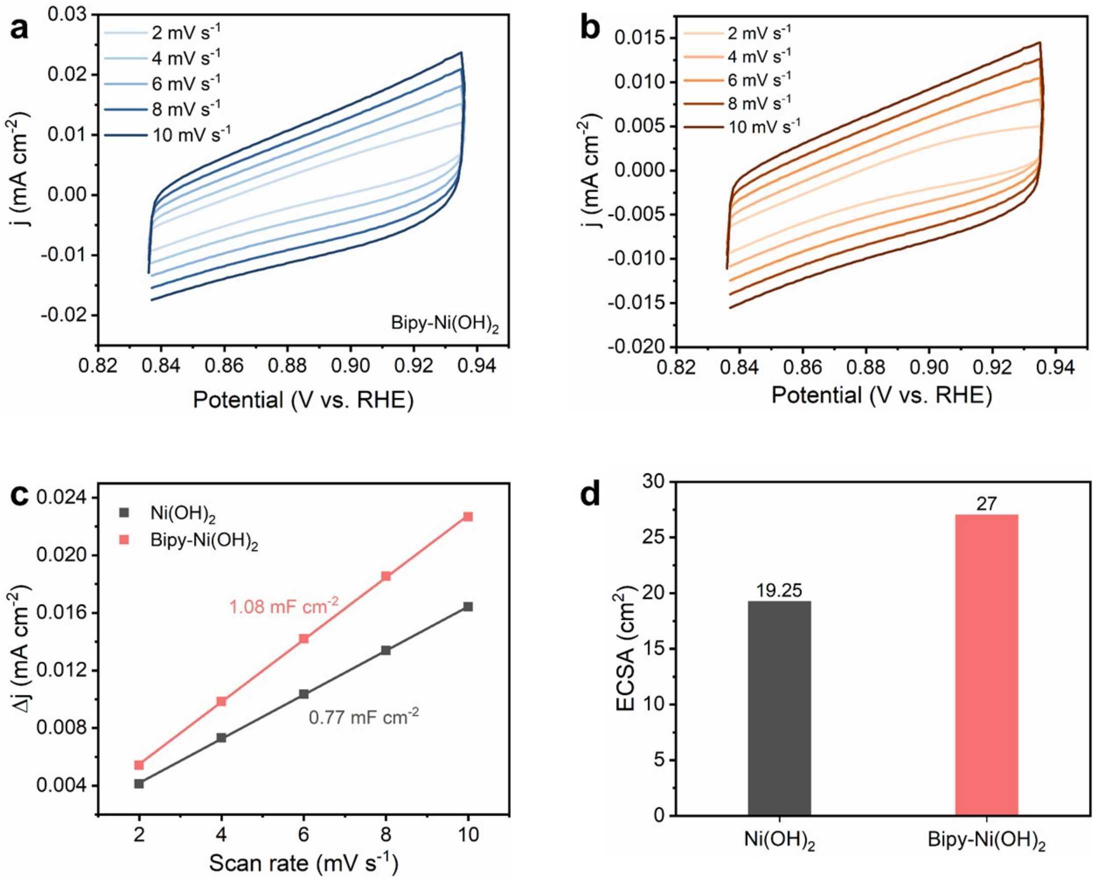

Figure S4. CV curves of (a) Bipy-$Ni(OH)_2$ and (b) $Ni(OH)_2$. (c) The C_{dl} values at the potential of 0.886 V vs. RHE, and (d) the corresponding ECSA values of $Ni(OH)_2$ and Bipy-$Ni(OH)_2$.

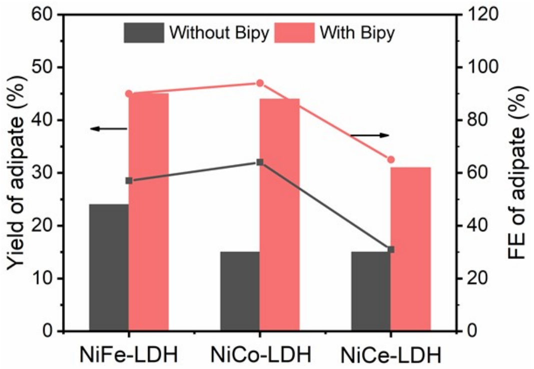

Figure S5. Adipate yield and FE over different Ni-based LDHs with and without Bipy modification. Electrolytic condition: 1.5 M NaOH with 0.1 M cyclohexanone at 1.53 V vs RHE for 2 h.

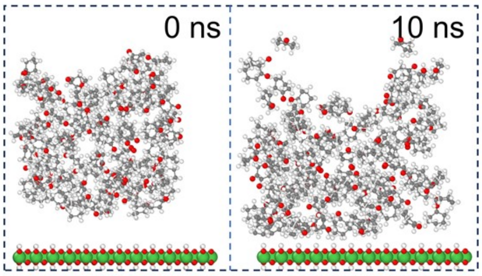

Figure S6. The snapshots from time-dependent MD simulations show the spatial distribution of cyclohexanone molecules near the pristine $Ni(OH)_2$ surfaces.

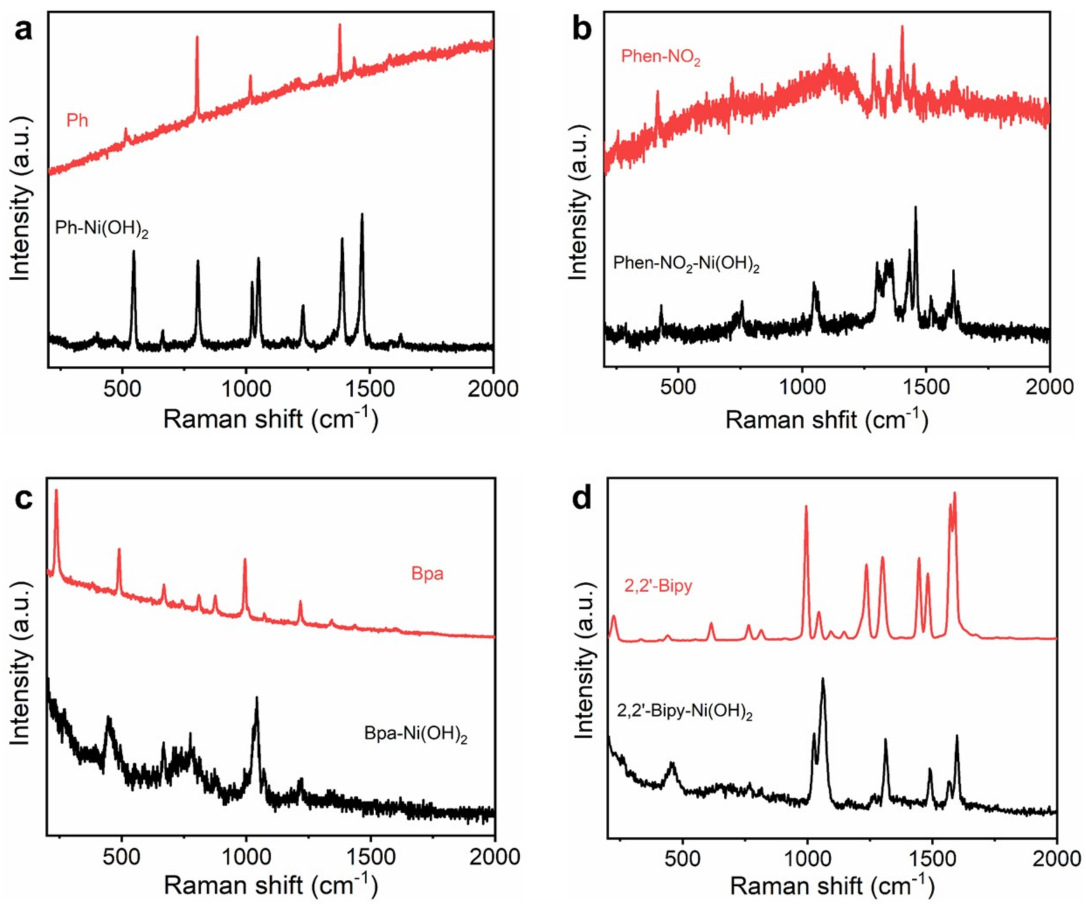

Figure S7. Raman spectroscopy of the N,N-containing conjugate ligands, including (a) Phen-NO_2, (b) Ph, (c) 2,2′-Bipy and (d) BPa, and the corresponding functionalized $Ni(OH)_2$ catalysts prepared via electrodeposition.

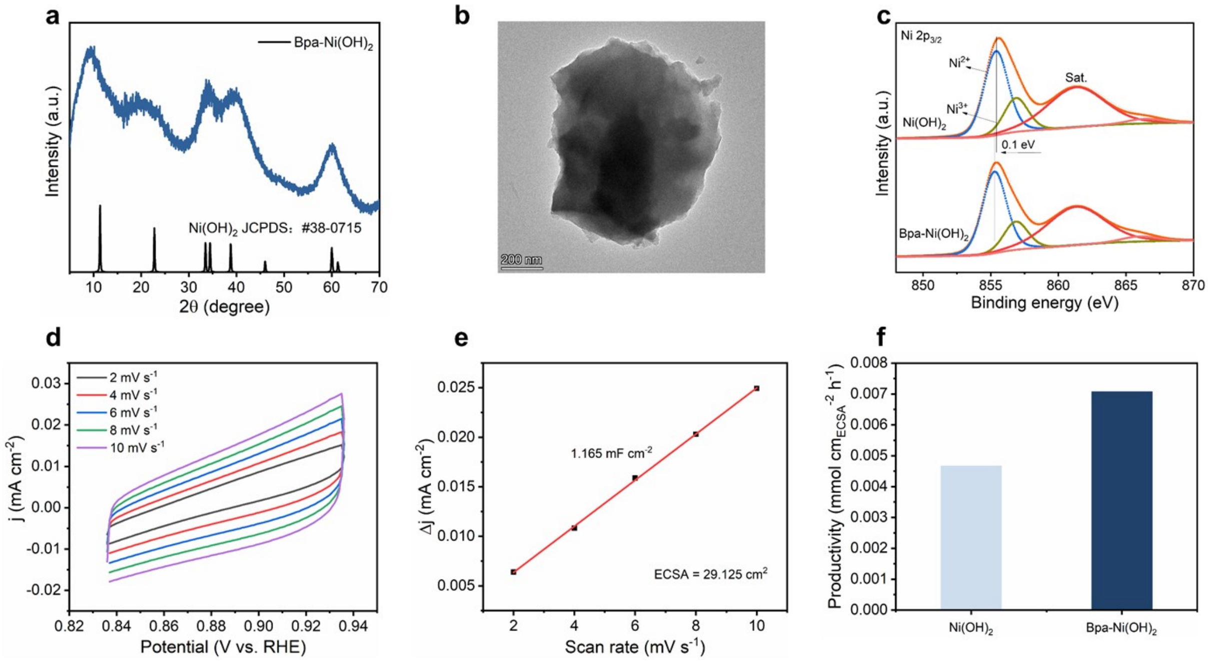

Figure S8. (a) XRD pattern and (b) TEM image of Bpa-$Ni(OH)_2$. (c) High-resolution XPS spectra showing the Ni $2p_{3/2}$ region for the pristine $Ni(OH)_2$ and Bpa-$Ni(OH)_2$ catalysts. (d) CV curves and (e) the corresponding C_{dl} value obtained through fitting for Bpa-$Ni(OH)_2$. (f) ECSA-normalized productivity of adipate for the pristine $Ni(OH)_2$ and Bpa-$Ni(OH)_2$ catalysts.

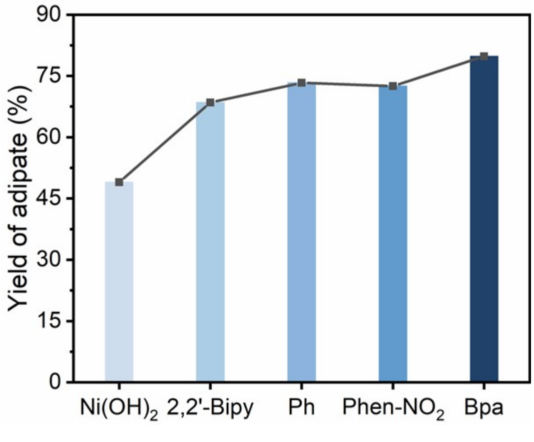

Figure S9. The yield of adipate of bare $Ni(OH)_2$ and ligand-functionalized $Ni(OH)_2$ after the electrolysis at 1.53 V vs. RHE for 10 h.

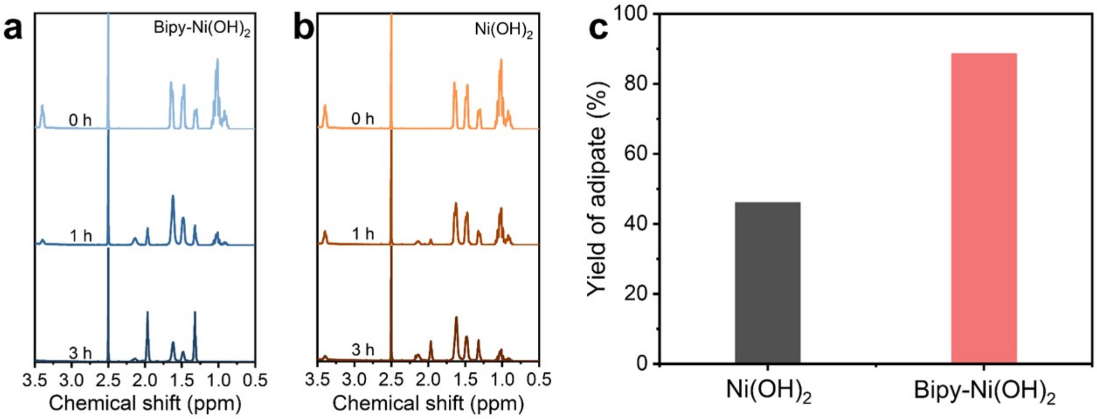

Figure S10. (a) ^{1}H NMR spectra of the reaction intermediates and products during the course of the cyclohexanol oxidation reaction at 1.53 V vs. RHE over $Ni(OH)_2$ and Bipy-$Ni(OH)_2$. (b) Comparison of the adipate yield after cyclohexanol electrolysis at 1.53 V vs. RHE for 14 h over $Ni(OH)_2$ and Bipy-$Ni(OH)_2$.

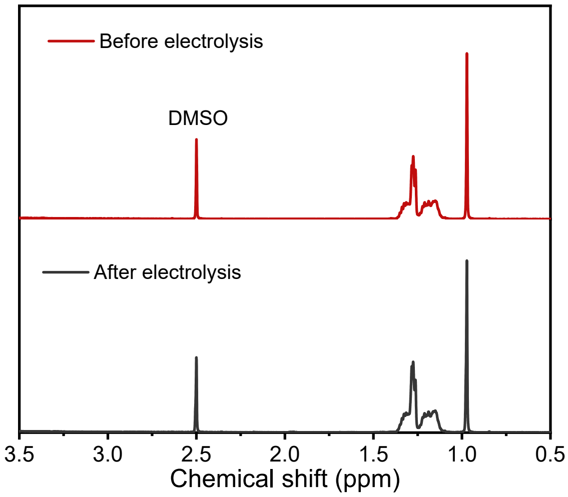

Figure S11. ^{1}H NMR spectra of 1-methylcyclohexanol before and after electrolysis, which showed no significant changes. Electrolytic condition: 0.1 M 1-methylcyclohexanol and 1.5 M NaOH, Bipy-$Ni(OH)_2$ as the catalysts, 1.53 V vs. RHE for 2 h.

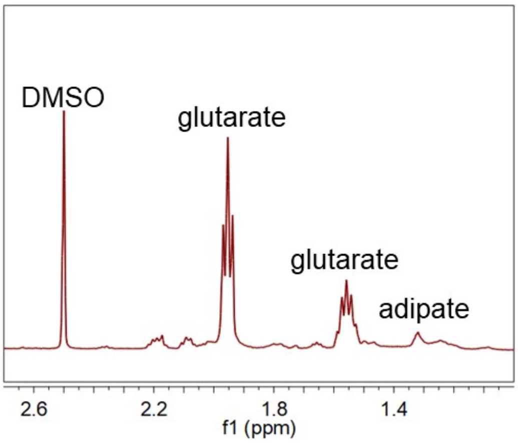

Figure S12. ^1H NMR spectra of 1,2-cyclohexanedione after electrolysis over Bipy-$Ni(OH)_2$ at 1.53 V vs. RHE for 2 h.

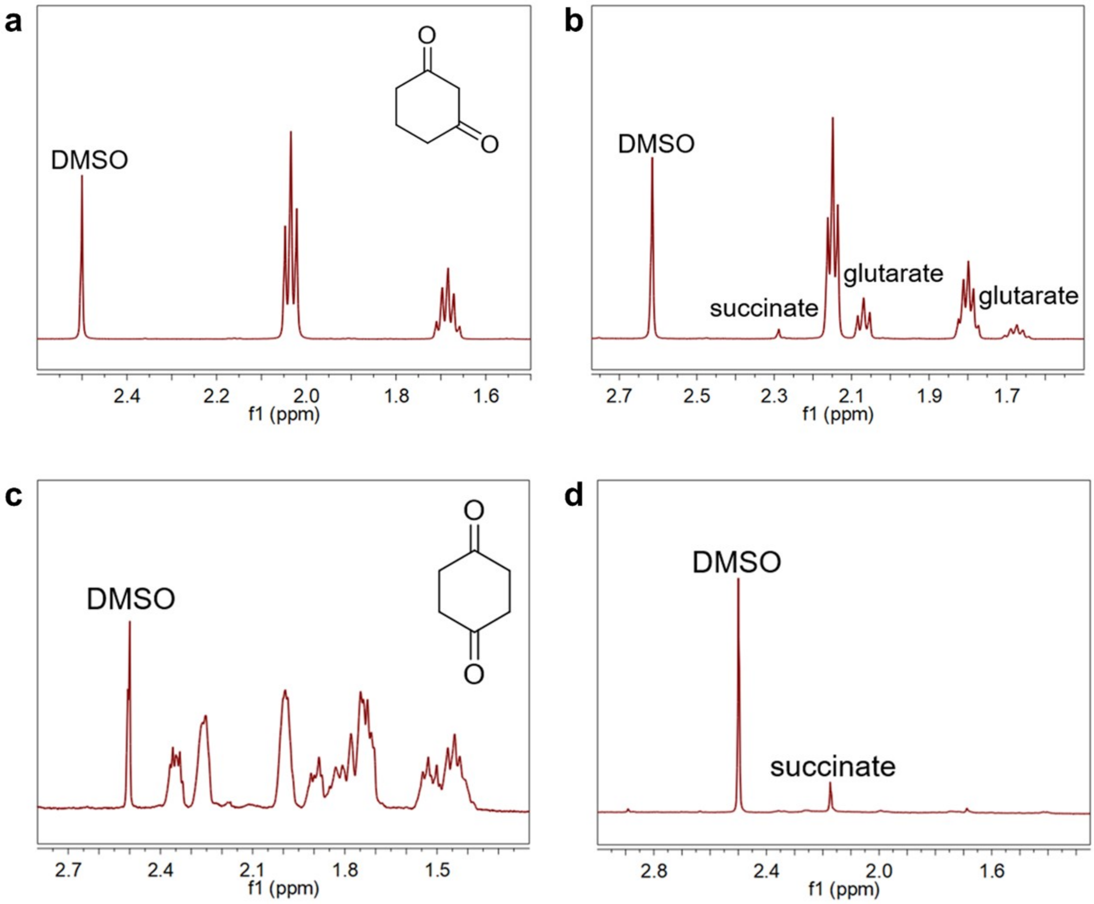

Figure S13. (a,c) ^1H NMR spectra of 1,3-cyclohexanedione and 1,4-cyclohexanedione in 1.5 M NaOH aqueous solutions. (b,d) ^1H NMR spectra of the products obtained using 1,3-cyclohexanedione and 1,4-cyclohexanedione as the substrate molecule after electrolysis over Bipy-$Ni(OH)_2$ at 1.53 V vs. RHE for 2 h.

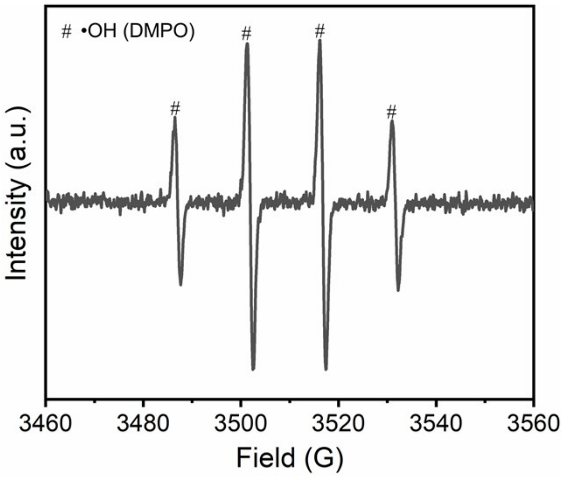

Figure S14. EPR spectrum of Bipy-$Ni(OH)_2$ monitored in 1.5 M NaOH in the absence of cyclohexanone.

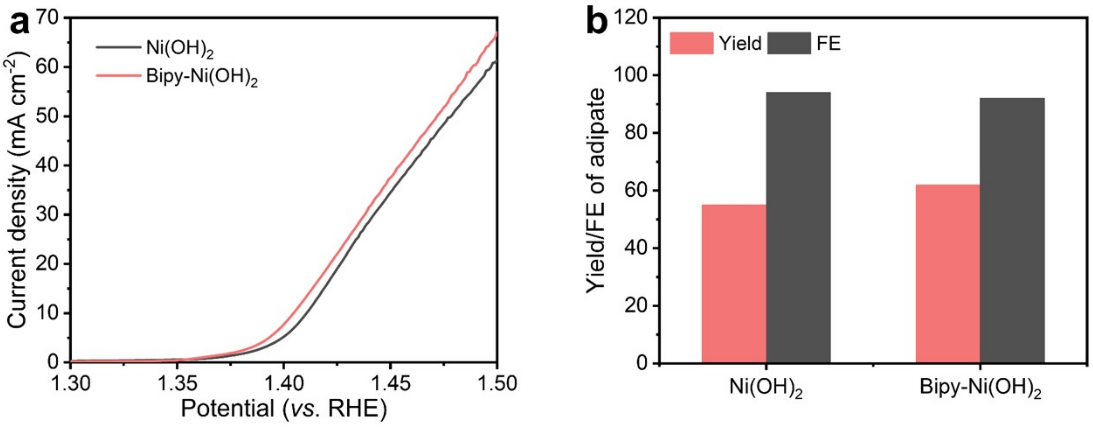

Figure S15. (a) Polarization curves of 1,6-hexanediol oxidation on the $Ni(OH)_2$ and Bipy-$Ni(OH)_2$ electrodes. (b) The yield and FE of adipate over $Ni(OH)_2$ and Bipy-$Ni(OH)_2$ at the potential of 1.53 V vs. RHE in 1.5 M NaOH with 0.1 M 1,6-hexanediol for 2 h.

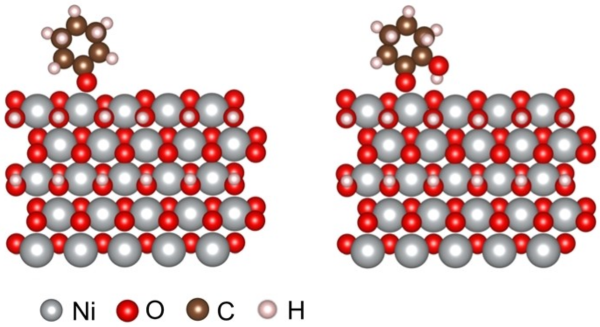

Figure S16. Optimized configuration of cyclohexanone (left) and 2-hydroxycyclohexanone (right) on bare $Ni(OH)_2$, respectively.

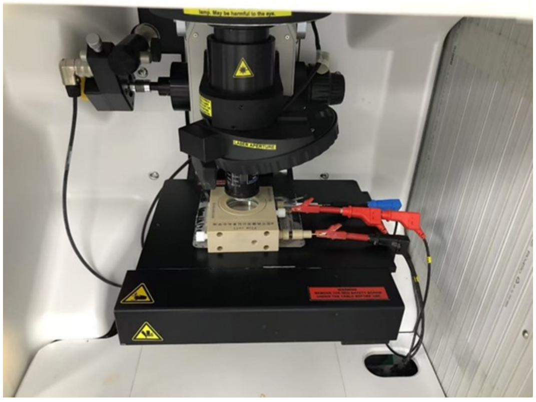

Figure S17. Photograph showing the in situ Raman test setup.

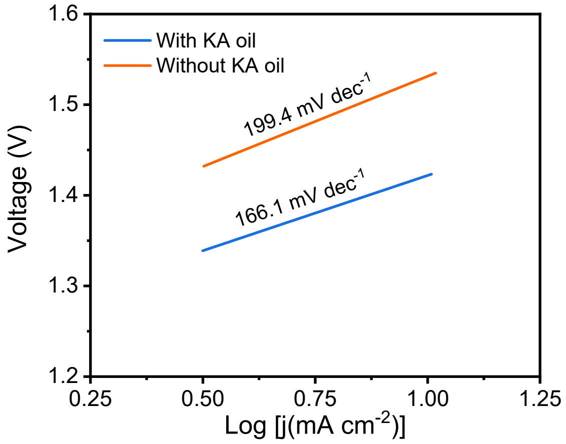

Figure S18. Tafel slope curves of the Bipy-$Ni(OH)_2$//Ru couple with and without KA oil.

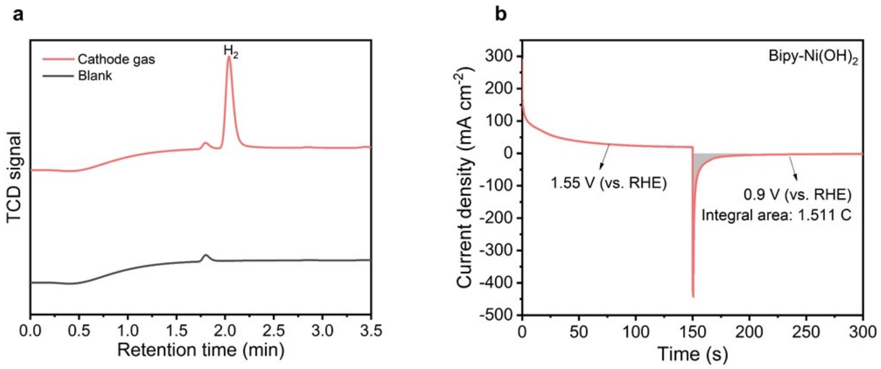

Figure S19. (a) GC data showing H_2 production at the Ru cathode. (b) Pulsed CA curves of Bipy-Ni(OH) by oxidization of the sample at 1.55 V vs RHE in 1.5 M NaOH for 150 s, followed by reduction at 0.9 V vs RHE for another 150 s.

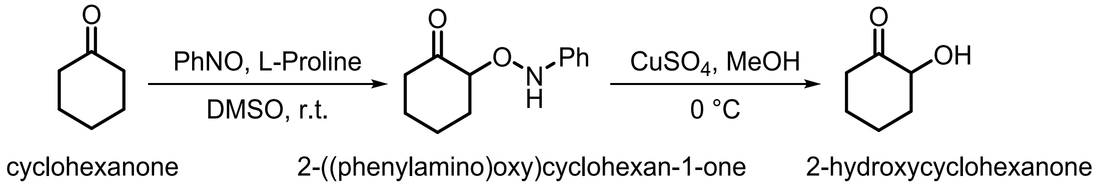

Scheme S1. Synthesis of 2-hydroxycyclohexanone.

SUPPLEMENTARY TABLES

Table S1. Comparison of the catalyst, electrolyte, reaction condition and electrochemical catalytic performance of cyclohexanol/cyclohexanone oxidation based on previous reports.

Entry	Literature	Catalyst	Electrolyte	Substrate	Reaction conditions	Yield (%)	Selectivity (%)
1	*Russian Chemical Bulletin, International Edition*, **2004**, 53, 688–692.	NiOOH	0.1 M NaOH	150 mM cyclohexanol	j_a = 6 mA cm^{-2}	46.7	N/A
2	*Russ. J. Appl. Chem.* **2014**, 87, 444–449.	NiOOH	1 M NaOH	150 mM cyclohexanol	j_a = 22 mA cm^{-2},with H_2O_2	50.2	89
3	*J. Solid State Electrochem.* **2016**, 20, 2773–2780.	Fe-Mn/Mn_2O_3	0.5 M NaOH	120 mM cyclohexanol	j_a = 100 mA cm^{-2}, 24 h, 60 °C	~83	N/A
4	*Angew. Chem. Int. Ed.* **2021**, 60, 8976–8982.	Mn-CoOOH	1 M KOH	20 mM cyclohexanol	1.45 V vs. RHE, 17 h	64.2	N/A
5	*Angew. Chem. Int. Ed.* **2022**, 61, e202214977.	Cu-$Ni(OH)_2$	1 M NaOH	100 mM cyclohexanol	1.63 V vs. RHE, 8 h	84	87
		Cu-$Ni(OH)_2$	1 M NaOH	100 mM cyclohexanone	1.63 V vs. RHE, 8 h	73	81
6	*Nat. Commun.* **2022**, 13, 5009.	SDS-$Ni(OH)_2$	0.5 M KOH	20 mM cyclohexanone	1.5 V vs. RHE, 16 h	84	N/A
		SDS-$Ni(OH)_2$	0.5 M KOH	20 mM cyclohexanol	1.5 V vs. RHE, 25 h	86.5	N/A
7	*J. Am. Chem. Soc.* **2024**, 146, 2, 1282–1293.	NiOOH	0.1 M NaOH	10 mM cyclohexanone	1.465 V vs. RHE, 6 h	93.6	95.7
8	*J. Am. Chem. Soc.* **2024**, 146, 15275–15285.	$CuCo_2O_4$	1 M KOH	150 mM cyclohexanol and cyclohexanone	j_a = 150 mA cm^{-2}	73	89
9	This work	Bipy-$Ni(OH)_2$	1.5 M NaOH	100 mM cyclohexanone	1.53 V vs. RHE, 10 h	90	91

Table S2. The electrolytic substrate molecules and corresponding major products using the Bipy-$Ni(OH)_2$ catalysts.

Entry	Structural formula	Major product
1	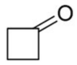	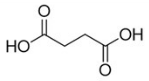
2	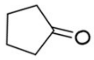	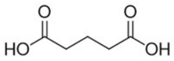
3	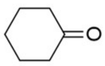	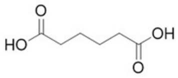
4	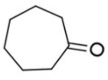	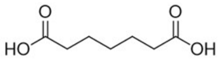
5	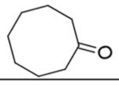	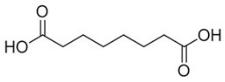

References

1. A. Bøgevig, H. Sundén, and A. Córdova, *Angew. Chem. Int. Ed.*, 2004, **43**, 1109–1112.
2. M. R. Morales, N. Momiyama, and H. Yamamoto, *Synlett*, 2006, **5**, 705-708.
3. H.-S. Yu, X.-J. Wei, J. Li, S.-Q. Gu, S. Zhang, L.-H. Wang, J.-Y. Ma, L.-N. Li, Q. Gao, R. Si, F.-F. Sun, Y. Wang, F. Song, H.-J. Xu, X.-H. Yu, Y. Zou, J.-Q. Wang, Z. Jiang, and Y.-Y. Huang, *Nucl. Sci. Tech.*, 2015, **26**, 050102.
4. B. Ravel, and M. Newville, *J. Synchrotron Radiat.*, 2005, **12**, 537–541.
5. G. Kresse, and J. Furthmüller, *Computat. Mater. Sci.*, 1996, **6**, 15–50.
6. G. Kresse, and J. Furthmüller, *Phys. Rev. B*, 1996, **54**, 11169–11186.
7. G. Kresse, and J. Hafner, *Phys. Rev. B*, 1993, **48**, 13115–13118.
8. J. P. Perdew, K. Burke, M. Ernzerhof, *Phys. Rev. Lett.*, 1996, **77**, 3865–3868.
9. P. E. Blöchl, *Phys. Rev. B*, 1994, **50**, 17953–17979.
10. G. Kresse, and D. Joubert, *Phys. Rev. B*, 1999, **59**, 1758–1775.
11. S. L. Dudarev, G. A. Botton, S. Y. Savrasov, C. J. Humphreys, and A. P. Sutton, *Phys. Rev. B*, 1998, **57**, 1505–1509.
12. J. K. Nørskov, J. Rossmeisl, A. Logadottir, L. Lindqvist, J. R. Kitchin, T. Bligaard, H. Jónsson, *J. Phys. Chem. B*, 2004, **108**, 17886–17892.
13. P. J. Linstrom, and W. G. Mallard, Eds., NIST Chemistry WebBook, NIST Standard Reference Database Number 69, National Institute of Standards and Technology, Gaithersburg MD, 20899, https://doi.org/10.18434/T4D303, (retrieved February 9, 2025).
14. S. Plimpton, *J. Comput. Phys.*, 1995, **117**, 1–19.
15. A. Stukowski, *Modelling Simul. Mater. Sci. Eng.*, 2009, **18**, 015012.
16. D. A. Case, H. M. Aktulga, K. Belfon, I. Y. Ben-Shalom, J. T. Berryman, S. R. Brozell, D. S. Cerutti, T. E. Cheatham, III, G. A. Cisneros, V. W. D. Cruzeiro, T. A. Darden, N. Forouzesh, M. Ghazimirsaeed, G. Giambaşu, T. Giese, M. K. Gilson, H. Gohlke, A. W. Goetz, J. Harris, Z. Huang, S. Izadi, S. A. Izmailov, K. Kasavajhala, M. C. Kaymak, A. Kovalenko, T. Kurtzman, T. S. Lee, P. Li, Z. Li, C. Lin, J. Liu, T. Luchko, R. Luo, M. Machado, M. Manathunga, K. M. Merz, Y. Miao, O. Mikhailovskii, G. Monard, H. Nguyen, K. A. O'Hearn, A. Onufriev, F. Pan, S. Pantano, A. Rahnamoun, D. R. Roe, A. Roitberg, C. Sagui, S. Schott-Verdugo, A. Shajan, J. Shen, C. L. Simmerling, N. R. Skrynnikov, J. Smith, J. Swails, R. C. Walker, J. Wang, J. Wang, X. Wu, Y. Wu, Y. Xiong, Y. Xue, D. M. York, C. Zhao, Q. Zhu, and P. A. Kollman, Amber 2024, University of California, San Francisco 2024.
17. M. P. Allen, and D. J. Tildesley, Computer simulation of liquids (2nd edn.) 2017, Oxford university press.
18. S. Nosé, *J. Chem. Phys.*, 1984, **81**, 511–519.
19. W. G. Hoover, *Phys. Rev. A*, 1985, **31**, 1695.
20. U. Essmann, L. Perera, M. L. Berkowitz, T. Darden, H. Lee, and L. G. Pedersen, *J. Chem. Phys.*, 1995, **103**, 8577–8593.
21. Z. Yan, H. Sun, X. Chen, H. Liu, Y. Zhao, H. Li, W. Xie, F. Cheng, and J. Chen, *Nat. Commun.*, 2018, **9**, 2373.
22. L. Peng, N. Yang, Y. Yang, Q. Wang, X. Xie, D. Sun-Waterhouse, L. Shang, T. Zhang, and G. I. N. Waterhouse, *Angew. Chem. Int. Ed.*, 2021, **60**, 24612–24619.

23. Y. Yang, C. Zhang, C. Zhang, Y. Shi, J. Li, B. Johannessen, Y. Liang, S. Zhang, Q. Song, H. Zhang, J. Huang, J. Ke, L. Zhang, Q. Song, J. Zeng, Y. Zhang, Z. Geng, P.-S. Wang, Z. Wang, J. Zeng, F. Li, *Nat. Commun.*, 2024, **15**, 6316.
24. Q. Zhang, P. Li, J. Wu, Y. Peng, and H. Pang, *Adv. Sci.*, 2023, **10**, 2304102.
25. J. Baltrusaitis, P.M. Jayaweera, and V.H. Grassian, *Phys. Chem. Chem. Phys.*, 2009, **11**, 8295–8305.
